# Exploring the Mechanisms of Arsenic Trioxide (*Pishuang*) in Hepatocellular Carcinoma Based on Network Pharmacology

**DOI:** 10.1155/2021/5773802

**Published:** 2021-11-29

**Authors:** Xinmiao Wang, Luchang Cao, Jingyuan Wu, Guanghui Zhu, Xiaoyu Zhu, Xiaoxiao Zhang, Duoduo Han, Ning Shui, Baoyi Ni, Jie Li

**Affiliations:** ^1^Guang'anmen Hospital, China Academy of Chinese Medical Sciences, Beijing 100053, China; ^2^Beijing University of Traditional Chinese Medicine, Beijing 100029, China

## Abstract

**Objective:**

Arsenic trioxide (*Pishuang*, Pishi, arsenolite, As_2_O_3_, and CAS 1327-53-3), a naturally occurring and toxic mineral as a drug for more than 2000 years in China, has been found to have a valuable function in hepatocellular carcinoma (HCC) in recent years. However, its exact mechanism remains to be elucidated. Therefore, this study was intended to explore the potential anti-HCC mechanism of arsenic trioxide through network pharmacology.

**Methods:**

The potential targets of arsenic trioxide were collected from PubChem and TargetNet. HCC targets were obtained from the GeneCards database. Then, a protein-protein interaction (PPI) network of arsenic trioxide and HCC common targets was established using STRING. GO and KEGG pathway enrichment analyses were performed by the Database for Annotation, Visualization, and Integrated Discovery (DAVID). Finally, an arsenic trioxide-target-pathway-HCC network was built by Cytoscape 3.2.1, and network topological analysis was carried out to screen the key candidate targets.

**Results:**

A total of 346 corresponding targets of arsenic trioxide and 521 HCC-related targets were collected. After target mapping, a total of 52 common targets were obtained. GO analysis showed that the biological process was mainly involved in the negative regulation of cellular senescence, response to tumor necrosis factor, and cellular response to hypoxia. Molecular functions included NF-kappa B binding, enzyme binding, p53 binding, and transcription factor binding. Cellular components mainly were replication fork, ESC/E(Z) complex, RNA polymerase II transcription factor complex, and organelle membrane. KEGG pathways were mainly enriched in the PI3K-Akt signaling pathway, VEGF signaling pathway, p53 signaling pathway, HIF-1 signaling pathway, TNF signaling pathway, AMPK signaling pathway, NF-kappa B signaling pathway, FoxO signaling pathway, ErbB signaling pathway, and MAPK signaling pathway. In the arsenic trioxide-target-pathway-HCC network, targets such as AKT1, RAF1, RELA, TP53, and PTEN had a higher degree*. Conclusions*. Our study showed that key targets of arsenic trioxide were mainly involved in multiple biological processes and pathways. It provided a theoretical basis for the screening of drug targets.

## 1. Introduction

Hepatocellular carcinoma (HCC) is a common malignant tumor with poor prognosis, characterized by strong invasion and rapid growth [1]. According to the latest data released by the International Agency for Research on Cancer (IARC), the incidence and mortality of HCC ranked 6th and 3rd among all cancers, respectively. The total number of new HCC cases worldwide was 905,677, accounting for 4.7% of all new cancer cases, while the number of HCC deaths was 830,180, accounting for 8.3% of all cancer deaths. The current therapies mainly include hepatic resection, liver transplantation, transarterial chemoembolization (TACE), and ablation. Meanwhile, the small-molecule targeted drugs such as sorafenib and lenvatinib and monoclonal antibodies such as nivolumab are also used for the systematic treatment of advanced HCC [2]. In spite of the development of modern medicine, the present therapeutic options of HCC are still limited. For example, all the registered studies including sorafenib failed to find any treatment to improve recurrence-free survival. In addition, tumor recurrence rate after surgery or ablation of HCC is as high as 70% [3]. Therefore, new anti-HCC therapies are urgently needed to improve the poor prognosis.

Nature is a rich source of natural products, among which minerals have attracted widespread attention in drug research and development because of their multitargeted activities and potential effect of anticancer. Arsenic trioxide (*Pishuang*, in Chinese Pinyin, As_2_O_3_, and CAS 1327-53-3), a natural and toxic substance be applied as a drug for more than 2000 years in China, has been found to have a valuable function in acute promyelocytic leukemia in recent years [4, 5]. Interestingly, studies have reported that arsenic trioxide has antineoplastic effects on HCC *in vitro* and *in vivo*. For instance, Bian et al. reported that the HCC patients who used arsenic trioxide transcatheter hepatic artery chemoembolization interventional therapy have a higher total effective rate and 1-year and 2-year survival rates than the control group (*P* < 0.05) [6]. Wang demonstrated that arsenic trioxide can inhibit proliferation and induce apoptosis of HCC cells through the ROS-mediated mitochondrial pathway [7]. Deng showed that arsenic trioxide could inhibit the growth of HCC subcutaneous transplanted tumors of nude mice and induce the apoptosis of hepatoma HepG2 cells [8]. Cai et al. proved that arsenic trioxide could induce upregulation of miR-1294 and suppress tumor growth in HCC cells by targeting TEAD1 and PIM1 [9]. Hu et al. reported that As_2_O_3_ nanoparticles could inhibit HCC tumor growth in the mice model, likely through downregulating PCNA- and DNMT-related proteins and upregulating GSDME-N [10]. Zhang et al. found that arsenic trioxide combined with canstatin could significantly inhibit HCC cell proliferation, migration, and adhesion abilities, promoted cell apoptosis, and inhibited tumor growth *in vitro* and *in vivo* [11]. It is observed that arsenic trioxide has a considerable prospect in the treatment of HCC. However, the exact mechanism underlying the therapeutic action of arsenic trioxide against HCC remains to be elucidated due to its character of multitarget and multipathway.

**Figure 1:**
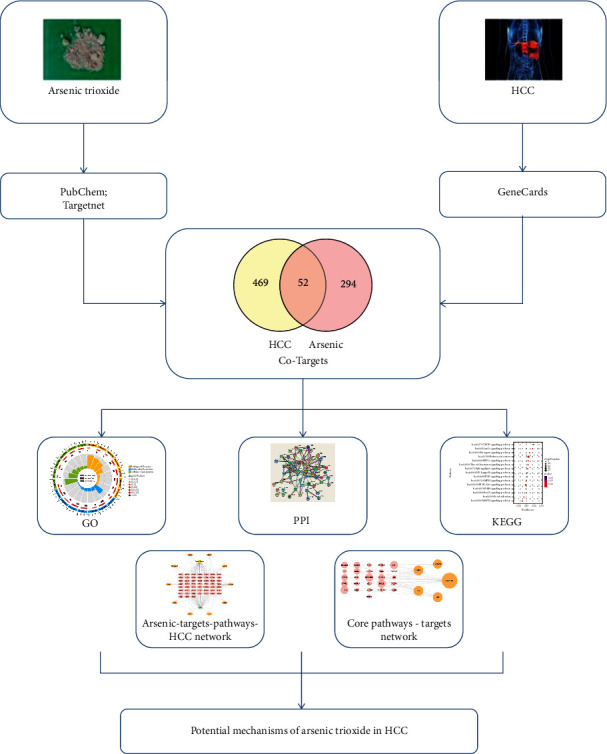
Workflow for arsenic trioxide against HCC.

Network pharmacology is a new discipline to explore the mechanism of drug efficacy [12]. It is a research method for designing multitarget drug molecules by selecting the key nodes in the network based on the theory of systems biology and network analysis. Not only does it benefit to the success rate of drug screening in clinical trials but also contribute to seeking for the most effective treatment for patients [13]. Therefore, this study was intended to explore the potential anti-HCC mechanism of arsenic trioxide through network pharmacology (Figure 1).

## 2. Materials and Methods

### 2.1. Collecting Targets of Arsenic Trioxide

We collected targets of arsenic trioxide by the following 3 ways: (1) by importing “CAS 1327-53-3” in PubChem [14] (https://pubchem.ncbi.nlm.nih.gov/), we collected targets' information (full name and gene symbol) of arsenic trioxide in “Chemical-Gene Co-Occurrences in Literature” and then applied UniProt [15] (https://www.uniprot.org/) to supplement their UniProt ID. (2) By inputting “CAS 1327-53-3” in PubChem, we obtained arsenic trioxide-related targets' information in “BioAssay Results” and used UniProt to collect their UniProt ID. (3) The arsenic trioxide-related targets' information was collected by inputting SMILES “O1[As]2O[As]3O[As]1O[As](O2)O3” in TargetNet [16] (http://targetnet.scbdd.com/). Targets in which prediction probability was 0 should be removed.

### 2.2. Collection of HCC-Related Targets

HCC-related targets were collected by retrieving “hepatocellular carcinoma” and “liver cancer” in GeneCards [17] (https://www.genecards.org/, version: 5.3). Only targets with “relevance score ≥20” could be included. The collected information included gene full name, gene symbol, and UniProt ID.

### 2.3. Construction of the Protein-Protein Interaction (PPI) Network

By mapping arsenic trioxide targets and HCC targets, we obtained arsenic trioxide and HCC common targets. Then, PPI network was constructed (combined score ≥0.9) through STRING [18] (http://string-db.org, version 11.0). The OmicShare tools (https://www.Omicshare.com/) were used to draw Venn diagrams.

### 2.4. GO and KEGG Analyses

Gene ontology (GO) and Kyoto Encyclopedia of Genes and Genomes (KEGG) analyses were performed by the Database for Annotation, Visualization, and Integrated Discovery [19] (DAVID, http://david.ncifcrf.Gov, version: 6.8). GO mainly included molecular function (MF), biological process (BP), and cellular component (CC). KEGG was used to identify the main anti-HCC signaling pathways of arsenic trioxide. In this study, we selected 20 GO terms and 10 KEGG pathways to be visualized by OmicShare tools.

### 2.5. Construction of the Arsenic Trioxide-Target-Pathway-HCC Network and Screening of Candidate Targets

The arsenic trioxide-target-pathway-HCC network was built by Cytoscape 3.2.1 [20] (http://www.cytoscape.org/). Network topological analysis was carried out to screen the key candidate targets. The topological parameters (degree, average shortest path length, closeness centrality, neighborhood connectivity, and radiality) of every node in the network were analysed, and all the results were ranked by “degree.”

## 3. Results

### 3.1. Collection of Arsenic Trioxide Targets

We obtained 34 targets in PubChem “Chemical-Gene Co-Occurrences in Literature,” 17 targets in PubChem “BioAssay Results,” and 310 targets collected from TargetNet. Finally, a total of 346 targets of arsenic trioxide were obtained after removing repeated targets (Supplementary Table S1).

### 3.2. Collection of HCC Targets

With the filtering criteria of “relevance score ≥20,” a total of 521 HCC-related targets were collected (Supplementary Table S2).

### 3.3. Construction of the PPI Network

**Figure 2:**
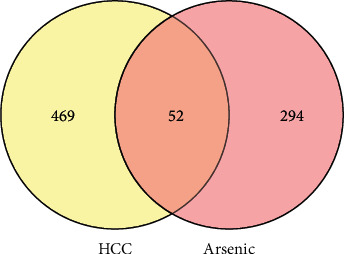
Venn diagram of arsenic trioxide (*Pishuang*) targets and HCC targets.

**Figure 3:**
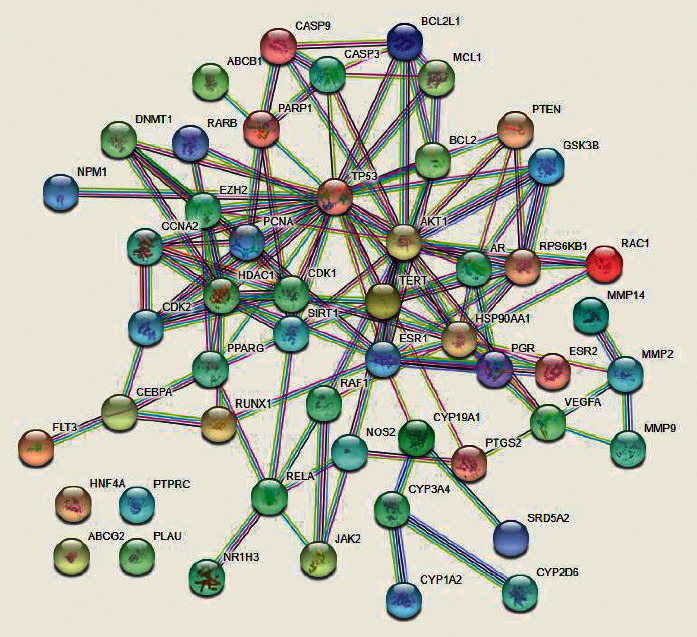
PPI network of common targets. Network nodes represent proteins. Edges represent protein-protein associations.

After mapping arsenic trioxide targets and HCC targets, a total of 52 common targets were obtained (Figure 2). To better understand the interactive relationships between the common targets, a PPI network (combined score ≥0.9) was built by STRING (Figure 3).

### 3.4. GO and KEGG Analyses

**Figure 4:**
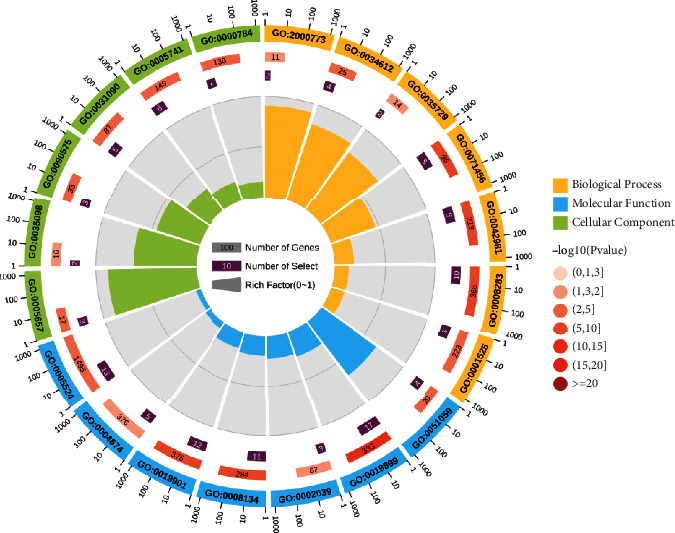
GO analysis. There are four circles in the figure. From outside to inside, the first circle is the classification of enrichment. Different colors represent different classifications. The second circle shows the number of background genes and *P* value. The more the genes, the longer the bars; the smaller the *P* value, redder the color. The third circle is the total number of prospective genes. The fourth circle represents rich factor, which indicates the ratio of genes in the current study versus the total genes in the term. GO:0071456: cellular response to hypoxia; GO:0008283: cell proliferation; GO:0042981: regulation of apoptotic process; GO:0034612: response to tumor necrosis factor; GO:0001525: angiogenesis; GO:2000773: negative regulation of cellular senescence; GO:0035729: cellular response to hepatocyte growth factor stimulus; GO:0005741: mitochondrial outer membrane; GO:0031090: organelle membrane; GO:0005657: replication fork; GO:0090575: RNA polymerase II transcription factor complex; GO:0000784: nuclear chromosome, telomeric region; GO:0035098: ESC/E(Z) complex; GO:0019899: enzyme binding; GO:0008134: transcription factor binding; GO:0019901: protein kinase binding; GO:0051059: NF-kappa B binding; GO:0005524: ATP binding; GO:0002039: p53 binding; GO:0004674: protein serine/threonine kinase activity.

GO analysis showed that the biological process was mainly involved in the negative regulation of cellular senescence, response to tumor necrosis factor, cellular response to hepatocyte growth factor stimulus, and cellular response to hypoxia. Molecular functions included NF-kappa B binding, enzyme binding, p53 binding, and transcription factor binding. Cellular components mainly were replication fork, ESC/E(Z) complex, RNA polymerase II transcription factor complex, and organelle membrane (Figure 4).

**Figure 5:**
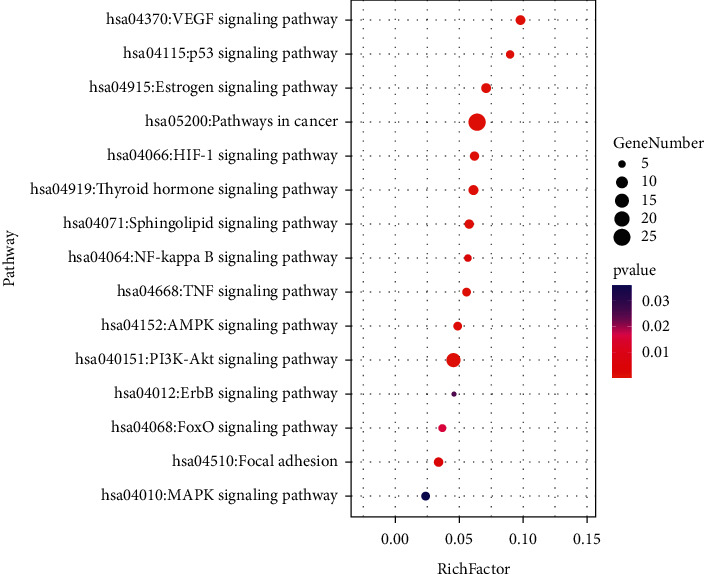
KEGG analysis. Node color is displayed in a gradient from red to green in the descending order of the *P* value. The size of the nodes is arranged in the ascending order according to the number of genes. Rich factor is the ratio of genes in the current study versus the total genes in the term.

There were 70 KEGG pathways obtained by DAVID, among which 59 pathways' *P* values were less than 0.05 (Supplementary Table S3). As shown in Figure 5, pathways closely associated with HCC included the VEGF signaling pathway, p53 signaling pathway, HIF-1 signaling pathway, NF-kappa B signaling pathway, TNF signaling pathway, AMPK signaling pathway, PI3K-Akt signaling pathway, ErbB signaling pathway, FoxO signaling pathway, MAPK signaling pathway, estrogen signaling pathway, pathways in cancer, thyroid hormone signaling pathway, focal adhesion, and sphingolipid signaling pathway.

### 3.5. Construction of the Arsenic Trioxide-Target-Pathway-HCC Network and Screening of Candidate Targets

**Figure 6:**
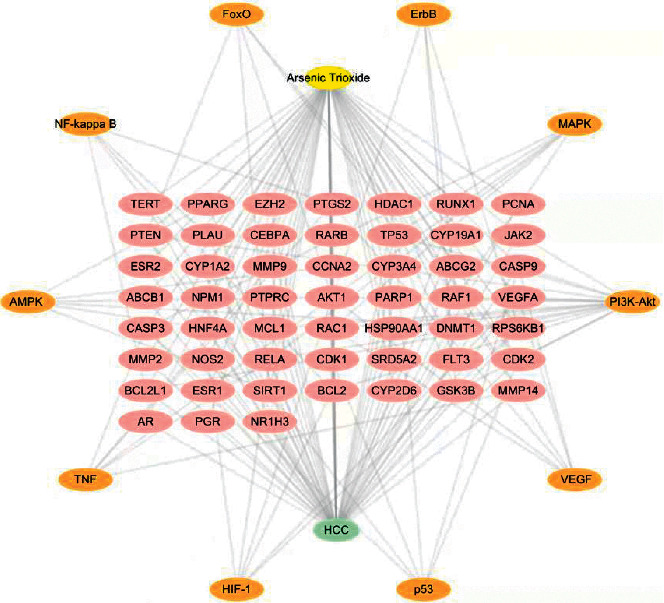
Arsenic trioxide-target-pathway-HCC network. Yellow node represents arsenic trioxide, green node represents HCC, pink nodes represent common targets, and orange nodes represent pathways.

Based on the common targets' and pathways' information, an arsenic trioxide-target-pathway-HCC network was constructed by Cytoscape 3.2.1 (Figure 6). The network topology analysis showed that targets such as AKT1, RAF1, RELA, RPS6KB1, TP53, and PTEN and pathways such as the PI3K-Akt signaling pathway, VEGF signaling pathway, p53 signaling pathway, and HIF-1 signaling pathway had a higher degree (Tables 1 and 2).

**Figure 7:**
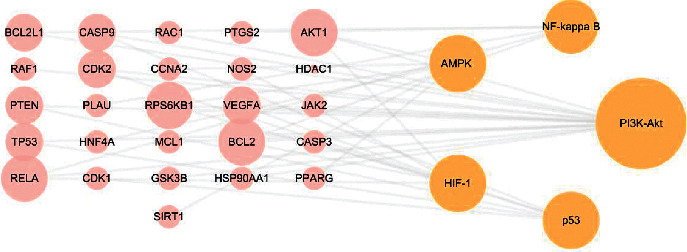
Core pathway-target network. Pink nodes represent targets, and orange nodes represent pathways. The nodes' size was determined by degree. The larger the node, the higher the degree.

Combining the research hotpots of HCC, we screened five pathways as the core pathways, and the relationships between each pathway and genes are shown in Figure 7.

## 4. Discussion

In this study, we obtained 346 targets of arsenic trioxide and 52 arsenic trioxide and HCC targets in common, which suggested that arsenic trioxide might be effective on HCC suppression by multitargets. As shown in the networks of arsenic trioxide-target-pathway-HCC and core pathway-target, nodes of AKT1, RAF1, TP53, and PTEN have a higher degree, indicating that they might be the key targets of arsenic trioxide for HCC therapy. Interestingly, AKT1 could regulate GSK-3*β* phosphorylation, another important target in the network and an isoform of GSK-3 which ubiquitously expressed serine/threonine kinase. Studies showed that regulating the Akt1/GSK-3*β* pathway could change HCC cell viability and migration [21]. Furthermore, it was reported that activation of GSK-3*β* contributed to arsenic trioxide-induced apoptosis in cancer cells [22]. As the significant marker in cancer treatment, RAF1 was found to overexpress in various cancers [23–25]. Accordingly, RAF-targeted small-molecule inhibitors have been applied in HCC and demonstrated high efficacy [26]. It was observed that the expression of RAF1 decreased following treatment with selective arsenic trioxide doses (1 *μ*M) [27]. The tumor suppressor PTEN is also a phosphoinositide phosphatase regulating the PI3K/Akt signaling pathway. Recent studies showed that PTEN mutations/deletions or low PTEN expression are closely related to HCC [28]. Goussetis and Platanias demonstrated that arsenic trioxide can induce upregulation of PTEN [29]. In the situation of TP53 mutation, cells with DNA damage can escape apoptosis and transform into cancer cells [30], which existed in at least 25% of HCC patients [31]. The high mutation rate of TP53 makes it to be a very promising potential therapeutic target. What is more exciting is that mutant p53 protein can be targeted by arsenic trioxide for degradation and plays a role in arsenic trioxide-mediated growth suppression [32].

GO analysis showed that the targets were mainly enriched in the regulation of cellular senescence, response to tumor necrosis factor, and hypoxia. Senescence is a cellular state in which cells lose their ability to proliferate [33]. A study showed that senescent cells could regulate immune cell activity and clear away atypical proliferative hepatocytes by secreting senescence-associated secretory phenotype factors [34]. Interestingly, Cheng et al. found that arsenic trioxide could induce a significant dose-related increase in the incidence of cellular senescence [35]. In addition, due to the imbalance between the rate of tumor cell proliferation and nutrient supply of vascular [36], hypoxia is a characteristic of solid tumors. Studies have shown that hypoxia occurred in the metastasis, poor prognosis, and radiation resistance of HCC [37, 38]. Arsenic trioxide could promote the apoptosis of cancer cells via hypoxia-inducible factor (HIF)-1*α* [39]. Most factors that regulate the progression of HCC are closely associated with inflammation. TNF-*α*, an important inflammatory mediator in immune responses, was demonstrated to induce tumor cell lysis [40, 41]. Furthermore, TNF-*α* expression was upregulated during the apoptosis of tumor cells by arsenic trioxide [42]. Notably, the results of KEGG pathway analysis were highly coincident with GO analysis. For example, HIFs are the “master” transcription factors responsible for gene expression in hypoxia [43]. Regulating the PI3K/Akt pathway and HIF-1*α* protein synthesis can inhibit hypoxia-induced angiogenesis and metastasis [44]. Mutant p53 induces a hypoxia transcriptional program in the tumor [45]. AMPK and NF-kappa B pathways were also found to participate in the inflammatory response during hypoxia and reoxygenation [46]. Accordingly, we speculate that arsenic trioxide may play an anti-HCC role through the regulation of cellular senescence, tumor necrosis factor, and hypoxia and is closely related to HIF, PI3K/Akt, p53, AMPK, and NF-kappa B pathways. In addition, although there is a lack of direct evidence that arsenic trioxide exerts anti-HCC effect by the estrogen signaling pathway, thyroid hormone signaling pathway, focal adhesion, and sphingolipid signaling pathway, studies have reported that these pathways are extremely related with HCC [47–50] and have higher rich factor in KEGG analysis, which indicates that these pathways might be the potential anti-HCC pathways of arsenic trioxide and points out the way for our future research.

In conclusion, we undertake a network pharmacology approach to explore the underlying anti-HCC molecular mechanisms of arsenic trioxide, which could provide a theoretical basis for the screening of drug targets. Our study showed that key targets of arsenic trioxide were mainly involved in multiple biological processes and pathways. In the near future, the series of promising results need to be verified by additional research, including *in vitro* and *in vivo* experiments.

## Figures and Tables

**Table 1:** Network topology analysis of targets (top 20 of degree).

No.	Targets	Degree	Average shortest path length	Closeness centrality	Neighborhood connectivity	Radiality
1	AKT1	10	1.873	0.534	15.900	0.782
2	RAF1	7	1.968	0.508	20.143	0.758
3	RELA	7	1.968	0.508	20.429	0.758
4	RPS6KB1	6	2.000	0.500	22.667	0.750
5	TP53	5	2.032	0.492	26.400	0.742
6	PTEN	5	2.032	0.492	26.200	0.742
7	VEGFA	5	2.032	0.492	26.400	0.742
8	CASP3	5	2.032	0.492	24.400	0.742
9	PTGS2	5	2.032	0.492	24.200	0.742
10	BCL2	5	2.032	0.492	26.200	0.742
11	CASP9	5	2.032	0.492	26.400	0.742
12	CDK2	5	2.032	0.492	26.200	0.742
13	RAC1	5	2.032	0.492	26.400	0.742
14	BCL2L1	4	2.063	0.485	31.250	0.734
15	GSK-3B	4	2.063	0.485	31.000	0.7341
16	SIRT1	4	2.063	0.485	28.750	0.7341
17	PPARG	3	2.095	0.477	36.667	0.726
18	JAK2	3	2.095	0.477	40.000	0.726
19	MMP9	3	2.095	0.477	36.667	0.726
20	HNF4A	3	2.095	0.477	36.667	0.726

**Table 2:** Network topology analysis of pathways.

No.	Entry ID	Pathway	Degree	Average shortest path length	Closeness centrality	Neighborhood connectivity	Radiality
1	hsa04151	PI3K-Akt signaling pathway	16	2.317	0.432	5.125	0.671
2	hsa04370	VEGF signaling pathway	6	2.635	0.380	6.167	0.591
3	hsa04115	p53 signaling pathway	6	2.762	0.362	4.667	0.560
4	hsa04066	HIF-1 signaling pathway	6	2.667	0.375	6.000	0.583
5	hsa04668	TNF signaling pathway	6	2.635	0.380	5.500	0.591
6	hsa04152	AMPK signaling pathway	6	2.698	0.371	4.833	0.575
7	hsa04010	MAPK signaling pathway	6	2.635	0.380	6.500	0.591
8	hsa04064	NF-kappa B signaling pathway	5	2.794	0.358	4.800	0.552
9	hsa04068	FoxO signaling pathway	5	2.698	0.371	6.200	0.575
10	hsa04012	ErbB signaling pathway	4	2.762	0.362	6.750	0.560

## Data Availability

The data used to support the results of this study can be obtained from the corresponding author upon reasonable request.

## Supplementary Materials

Supplementary Materials Supplementary Table S1: arsenic trioxide targets. Supplementary Table S2: hepatocellular carcinoma targets. Supplementary Table S3: KEGG pathways (*P* < 0.05).
