# Supplementary material for: Exploring the Mechanisms of Arsenic Trioxide (Pishuang) in Hepatocellular Carcinoma Based on Network Pharmacology
Source: Evid Based Complement Alternat Med. 2021 Nov 29;2021:5773802. doi: 10.1155/2021/5773802 (PMC8648446; doi:10.1155/2021/5773802)
Supplement: Supplementary Materials — Supplementary Table S1: arsenic trioxide targets. Supplementary Table S2: hepatocellular carcinoma targets. Supplementary Table S3: KEGG pathways (P < 0.05). [file 5773802.f1.zip › 5773802.f1/Supplementary Table S1 (1).pdf]

Supplementary Table S1. Arsenic trioxide targets

Uniprot ID	Gene name	Gene symbol
P12268	Inosine-5'-monophosphate dehydrogenase 2	IMPDH2
P30543	Adenosine receptor A2a	Adora2a
P05129	Protein kinase C gamma type	PRKCG
O00311	Cell division cycle 7-related protein kinase	CDC7
P60953	Cell division control protein 42 homolog	CDC42
P18031	Tyrosine-protein phosphatase non-receptor type	PTPN1
P04049	RAF proto-oncogene serine/threonine-protein kinase	RAF1
Q13946	High affinity cAMP-specific 3',5'-cyclic phosphodiesterase 7A	PDE7A
P42338	Phosphatidylinositol 4,5-bisphosphate 3-kinase catalytic subunit beta isoform	PIK3CB
Q08499	cAMP-specific 3',5'-cyclic phosphodiesterase 4D	PDE4D
P28190	Adenosine receptor A1	ADORA1
P25099	Adenosine receptor A1	Adora1
P07900	Heat shock protein HSP 90-alpha	HSP90AA1
Q9NWZ3	Interleukin-1 receptor-associated kinase 4	IRAK4
P25105	Platelet-activating factor receptor	PTAFR
P16116	Aldose reductase	AKR1B1
P23795	Acetylcholinesterase	ACHE
P41235	Hepatocyte nuclear factor 4-alpha	HNF4A
Q14432	cGMP-inhibited 3',5'-cyclic phosphodiesterase A	PDE3A
P11712	Cytochrome P450 2C9	CYP2C9
P11309	Serine/threonine-protein kinase pim-1	PIM1
P21554	Cannabinoid receptor 1	CNR1
O60674	Tyrosine-protein kinase JAK2	JAK2
P23443	Ribosomal protein S6 kinase beta-1	RPS6KB1
P51812	Ribosomal protein S6 kinase alpha-3	RPS6KA3
P08235	Mineralocorticoid receptor	NR3C2
P30939	5-hydroxytryptamine receptor 1F	HTR1F
Q07820	Induced myeloid leukemia cell differentiation protein Mcl-1	MCL1
P80365	Corticosteroid 11-beta-dehydrogenase isozyme 2	HSD11B2
Q8TDU6	G-protein coupled bile acid receptor 1	GPBAR1
P49759	Dual specificity protein kinase CLK1	CLK1
O42275	Acetylcholinesterase	ache
P00491	Purine nucleoside phosphorylase	PNP
P05622	Platelet-derived growth factor receptor beta	Pdgfrb
P31421	Metabotropic glutamate receptor 2	Grm2
P08311	Cathepsin G	CTSG
P49841	Glycogen synthase kinase-3 beta	GSK3B
Q13133	Oxysterols receptor LXR-alpha	NR1H3
P37136	Acetylcholinesterase	Ache
P20272	Cannabinoid receptor 1	Cnr1
P06401	Progesterone receptor	PGR

Q13627	Dual specificity tyrosine-phosphorylation-regulated kinase 1A	DYRK1A
P02550	Tubulin alpha-1A chain	TUBA1A
P34972	Cannabinoid receptor 2	CNR2
P35236	Tyrosine-protein phosphatase non-receptor type	PTPN7
P63000	Ras-related C3 botulinum toxin substrate 1	RAC1
P21728	D(1A) dopamine receptor	DRD1
P08575	Receptor-type tyrosine-protein phosphatase C	PTPRC
P47936	Cannabinoid receptor 2	Cnr2
Q05469	Hormone-sensitive lipase	LIPE
P23385	Metabotropic glutamate receptor 1	Grm1
P49840	Glycogen synthase kinase-3 alpha	GSK3A
P15121	Aldose reductase	AKR1B1
Q9Y5X4	Photoreceptor-specific nuclear receptor	NR2E3
O60240	Perilipin-1	PLIN1
P26358	DNA (cytosine-5)-methyltransferase 1	DNMT1
P05364	Beta-lactamase	ampC
P04054	Phospholipase A2	PLA2G1B
O70536	Sterol O-acyltransferase 1	Soat1
Q05769	Prostaglandin G/H synthase 2	Ptgs2
Q86TI2	Dipeptidyl peptidase 9	DPP9
Q00G26	Perilipin-5	PLIN5
Q9QZN9	Cannabinoid receptor 2	Cnr2
P68400	Casein kinase II subunit alpha	CSNK2A1
Q9BY41	Histone deacetylase 8	HDAC8
P08473	Neprilysin	MME
P08183	Multidrug resistance protein 1	ABCB1
O35433	Transient receptor potential cation channel subfamily V member 1	Trpv1
P51677	C-C chemokine receptor type 3	CCR3
P07858	Cathepsin B	CTSB
P10827	Thyroid hormone receptor alpha	THRA
P10826	Retinoic acid receptor beta	RARB
P10276	Retinoic acid receptor alpha	RARA
P19327	5-hydroxytryptamine receptor 1A	Htr1a
P35367	Histamine H1 receptor	HRH1
Q15722	Leukotriene B4 receptor 1	LTB4R
P43140	Alpha-1A adrenergic receptor	Adra1a
P49286	Melatonin receptor type 1B	MTNR1B
P09960	Leukotriene A-4 hydrolase	LTA4H
P12821	Angiotensin-converting enzyme	ACE
P00750	Tissue-type plasminogen activator	PLAT
P32240	Prostaglandin E2 receptor EP4 subtype	Ptger4
P13631	Retinoic acid receptor gamma	RARG
O43497	Voltage-dependent T-type calcium channel subunit alpha-1G	CACNA1G
P21917	D(4) dopamine receptor	DRD4

P43088	Prostaglandin F2-alpha receptor	PTGFR
P48147	Prolyl endopeptidase	PREP
P35610	Sterol O-acyltransferase 1	SOAT1
O60725	Protein-S-isoprenylcysteine O-methyltransferase	ICMT
P08588	Beta-1 adrenergic receptor	ADRB1
Q62053	Prostaglandin E2 receptor EP2 subtype	Ptger2
Q95323	Carbonic anhydrase 4	CA4
Q13464	Rho-associated protein kinase 1	ROCK1
Q62758	5-hydroxytryptamine receptor 4	Htr4
P61169	D(2) dopamine receptor	Drd2
P28221	5-hydroxytryptamine receptor 1D	HTR1D
P14416	D(2) dopamine receptor	DRD2
P52732	Kinesin-like protein KIF11	KIF11
P30557	Prostaglandin E2 receptor EP3 subtype	Ptger3
Q5RAG0	Histone deacetylase 1	HDAC1
P00749	Urokinase-type plasminogen activator	PLAU
P48039	Melatonin receptor type 1A	MTNR1A
P23978	Sodium- and chloride-dependent GABA transporter 1	Slc6a1
O15379	Histone deacetylase 3	HDAC3
P32246	C-C chemokine receptor type 1	CCR1
P43116	Prostaglandin E2 receptor EP2 subtype	PTGER2
Q00975	Voltage-dependent N-type calcium channel subunit alpha-1B	CACNA1B
P08254	Stromelysin-1	MMP3
Q64663	P2X purinoceptor 7	P2rx7
P34969	5-hydroxytryptamine receptor 7	HTR7
P55211	Caspase-9	CASP9
P31648	Sodium- and chloride-dependent GABA transporter 1	Slc6a1
P14780	Matrix metalloproteinase-9	MMP9
Q16548	Bcl-2-related protein A1	BCL2A1
P13945	Beta-3 adrenergic receptor	ADRB3
O95822	Malonyl-CoA decarboxylase, mitochondrial	MLYCD
P48067	Sodium- and chloride-dependent glycine transporter 1	SLC6A9
P08514	Integrin alpha-IIb	ITGA2B
Q13547	Histone deacetylase 1	HDAC1
P32305	5-hydroxytryptamine receptor 7	Htr7
P39900	Macrophage metalloelastase	MMP12
P24557	Thromboxane-A synthase	TBXAS1
P14061	Estradiol 17-beta-dehydrogenase 1	HSD17B1
P50130	D(1A) dopamine receptor	DRD1
P19020	D(3) dopamine receptor	Drd3
P07861	Neprilysin	Mme
O14842	Free fatty acid receptor 1	FFAR1
Q15661	Tryptase alpha/beta-1	TPSAB1

P21836	Acetylcholinesterase	Ache
P49430	Thromboxane-A synthase	Tbxas1
O75116	Rho-associated protein kinase 2	ROCK2
Q92769	Histone deacetylase 2	HDAC2
P42330	Aldo-keto reductase family 1 member C3	AKR1C3
P43235	Cathepsin K	CTSK
Q9UBN7	Histone deacetylase 6	HDAC6
P48443	Retinoic acid receptor RXR-gamma	RXRG
Q63470	Dual specificity tyrosine-phosphorylation-regulated kinase 1A	Dyrk1a
P20231	Tryptase beta-2	TPSB2
P41597	C-C chemokine receptor type 2	CCR2
P07384	Calpain-1 catalytic subunit	CAPN1
P09958	Furin	FURIN
P18405	3-oxo-5-alpha-steroid 4-dehydrogenase 1	SRD5A1
Q99572	P2X purinoceptor 7	P2RX7
P09917	Arachidonate 5-lipoxygenase	ALOX5
P08908	5-hydroxytryptamine receptor 1A	HTR1A
P20648	Potassium-transporting ATPase alpha chain 1	ATP4A
P21453	Sphingosine 1-phosphate receptor 1	S1PR1
Q6V1X1	Dipeptidyl peptidase 8	DPP8
Q00960	Glutamate receptor ionotropic, NMDA 2B	Grin2b
P07711	Cathepsin L1	CTSL
O00519	Fatty-acid amide hydrolase 1	FAAH
P97612	Fatty-acid amide hydrolase 1	Faah
P31213	3-oxo-5-alpha-steroid 4-dehydrogenase 2	SRD5A2
P27487	Dipeptidyl peptidase 4	DPP4
P07478	Trypsin-2	PRSS2
P08483	Muscarinic acetylcholine receptor M3	Chrm3
P08842	Steryl-sulfatase	STS
Q13224	Glutamate receptor ionotropic, NMDA 2B	GRIN2B
P34913	Bifunctional epoxide hydrolase 2	EPHX2
P20309	Muscarinic acetylcholine receptor M3	CHRM3
P12822	Angiotensin-converting enzyme	ACE
P31652	Sodium-dependent serotonin transporter	Slc6a4
P31645	Sodium-dependent serotonin transporter	SLC6A4
Q14524	Sodium channel protein type 5 subunit alpha	SCN5A
Q95136	D(1A) dopamine receptor	DRD1
Q02769	Squalene synthase	Fdft1
Q9H228	Sphingosine 1-phosphate receptor 5	S1PR5
P28223	5-hydroxytryptamine receptor 2A	HTR2A
Q96EB6	NAD-dependent protein deacetylase sirtuin-1	SIRT1
P08172	Muscarinic acetylcholine receptor M2	CHRM2
P37059	Estradiol 17-beta-dehydrogenase 2	HSD17B2
Q04609	Glutamate carboxypeptidase 2	FOLH1
P35439	Glutamate receptor ionotropic, NMDA 1	Grin1
Q9UNQ0	ATP-binding cassette sub-family G member 2	ABCG2

P25021	Histamine H2 receptor	HRH2
O14684	Prostaglandin E synthase	PTGES
P31424	Metabotropic glutamate receptor 5	Grm5
P30729	D(4) dopamine receptor	Drd4
Q9Y5N1	Histamine H3 receptor	HRH3
P18901	D(1A) dopamine receptor	Drd1
P28564	5-hydroxytryptamine receptor 1B	Htr1b
P13922	Bifunctional dihydrofolate reductase-thymidylate synthase	——
P35563	5-hydroxytryptamine receptor 3A	Htr3a
P50579	Methionine aminopeptidase 2	METAP2
Q9QYN8	Histamine H3 receptor	Hrh3
P63001	Ras-related C3 botulinum toxin substrate 1	Rac1
P19099	Cytochrome P450 11B2, mitochondrial	CYP11B2
Q60492	Sigma non-opioid intracellular receptor 1	SIGMAR1
P10635	Cytochrome P450 2D6	CYP2D6
O14746	Telomerase reverse transcriptase	TERT
P19634	Sodium/hydrogen exchanger 1	SLC9A1
P50172	Corticosteroid 11-beta-dehydrogenase isozyme 1	Hsd11b1
Q3KRE8	Tubulin beta-2B chain	Tubb2b
P33261	Cytochrome P450 2C19	CYP2C19
P25779	Cruzipain	——
P15144	Aminopeptidase N	ANPEP
P23977	Sodium-dependent dopamine transporter	Slc6a3
P31389	Histamine H1 receptor	HRH1
P15538	Cytochrome P450 11B1, mitochondrial	CYP11B1
P08913	Alpha-2A adrenergic receptor	ADRA2A
P08912	Muscarinic acetylcholine receptor M5	CHRM5
P15917	Lethal factor	lef
Q14416	Metabotropic glutamate receptor 2	GRM2
Q6B856	Tubulin beta-2B chain	TUBB2B
P11229	Muscarinic acetylcholine receptor M1	CHRM1
P22303	Acetylcholinesterase	ACHE
Q8IXJ6	NAD-dependent protein deacetylase sirtuin-2	SIRT2
O95180	Voltage-dependent T-type calcium channel subunit alpha-1H	CACNA1H
P18130	Alpha-1A adrenergic receptor	ADRA1A
Q9UHL4	Dipeptidyl peptidase 2	DPP7
O60755	Galanin receptor type 3	GALR3
P14842	5-hydroxytryptamine receptor 2A	Htr2a
P28845	Corticosteroid 11-beta-dehydrogenase isozyme 1	HSD11B1
P18825	Alpha-2C adrenergic receptor	ADRA2C
P36544	Neuronal acetylcholine receptor subunit alpha-7	CHRNA7
P56524	Histone deacetylase 4	HDAC4
Q9JI35	Histamine H3 receptor	HRH3
P28335	5-hydroxytryptamine receptor 2C	HTR2C

Q9GZU7	Carboxy-terminal domain RNA polymerase II polypeptide A small phosphatase 1	CTDSP1
P31388	5-hydroxytryptamine receptor 6	Htr6
P42262	Glutamate receptor 2	GRIA2
Q923Y8	Trace amine-associated receptor 1	Taar1
O95977	Sphingosine 1-phosphate receptor 4	S1PR4
P23975	Sodium-dependent noradrenaline transporter	SLC6A2
P20288	D(2) dopamine receptor	DRD2
P24468	COUP transcription factor 2	NR2F2
P31390	Histamine H1 receptor	Hrh1
Q05941	Neuronal acetylcholine receptor subunit alpha-7	Chrna7
P80457	Xanthine dehydrogenase/oxidase	XDH
Q9HC97	G-protein coupled receptor 35	GPR35
P35354	Prostaglandin G/H synthase 2	PTGS2
Q01959	Sodium-dependent dopamine transporter	SLC6A3
Q494W8	CHRNA7-FAM7A fusion protein	CHRFAM7A
P21918	D(1B) dopamine receptor	DRD5
O43353	Receptor-interacting serine/threonine-protein kinase 2	RIPK2
Q92731	Estrogen receptor beta	ESR2
P51452	Dual specificity protein phosphatase 3	DUSP3
Q9R0C9	Sigma non-opioid intracellular receptor 1	Sigmar1
Q96RJ0	Trace amine-associated receptor 1	TAAR1
P05067	Amyloid beta A4 protein	APP
P00918	Carbonic anhydrase 2	CA2
Q9H3N8	Histamine H4 receptor	HRH4
P79208	Prostaglandin G/H synthase 2	PTGS2
P05177	Cytochrome P450 1A2	CYP1A2
P18089	Alpha-2B adrenergic receptor	ADRA2B
Q8TDS4	Hydroxycarboxylic acid receptor 2	HCAR2
Q04206	Transcription factor p65	RELA
P10980	Muscarinic acetylcholine receptor M2	Chrm2
P35218	Carbonic anhydrase 5A, mitochondrial	CA5A
P27338	Amine oxidase [flavin-containing] B	MAOB
P19643	Amine oxidase [flavin-containing] B	Maob
O43570	Carbonic anhydrase 12	CA12
P05093	Steroid 17-alpha-hydroxylase/17,20 lyase	CYP17A1
Q9ULX7	Carbonic anhydrase 14	CA14
P29476	Nitric oxide synthase, brain	Nos1
P29477	Nitric oxide synthase, inducible	Nos2
P29474	Nitric oxide synthase, endothelial	NOS3
P29475	Nitric oxide synthase, brain	NOS1
P21397	Amine oxidase [flavin-containing] A	MAOA
P21396	Amine oxidase [flavin-containing] A	Maoa
P05979	Prostaglandin G/H synthase 1	PTGS1
Q9HC16	DNA dC->dU-editing enzyme APOBEC-3G	APOBEC3G
P23219	Prostaglandin G/H synthase 1	PTGS1

P35228	Nitric oxide synthase, inducible	NOS2
Q16790	Carbonic anhydrase 9	CA9
P14174	Macrophage migration inhibitory factor	MIF
O42713	Polyphenol oxidase 2	PPO2
P04058	Acetylcholinesterase	ache
P15207	Androgen receptor	Ar
P16050	Arachidonate 15-lipoxygenase	ALOX15
P28566	5-hydroxytryptamine receptor 1E	HTR1E
P23141	Liver carboxylesterase 1	CES1
P31941	DNA dC->dU-editing enzyme APOBEC-3A	APOBEC3A
P08173	Muscarinic acetylcholine receptor M4	CHRM4
P47898	5-hydroxytryptamine receptor 5A	HTR5A
Q99720	Sigma non-opioid intracellular receptor 1	SIGMAR1
P08482	Muscarinic acetylcholine receptor M1	Chrm1
O95136	Sphingosine 1-phosphate receptor 2	S1PR2
P08909	5-hydroxytryptamine receptor 2C	Htr2c
P09483	Neuronal acetylcholine receptor subunit alpha-4	Chrna4
O00748	Cocaine esterase	CES2
P00915	Carbonic anhydrase 1	CA1
P35398	Nuclear receptor ROR-alpha	RORA
P43166	Carbonic anhydrase 7	CA7
P14324	Farnesyl pyrophosphate synthase	FDPS
P22086	Alpha-2C adrenergic receptor	Adra2c
Q99685	Monoglyceride lipase	MGLL
P12527	Arachidonate 5-lipoxygenase	Alox5
P11086	Phenylethanolamine N-methyltransferase	PNMT
P07943	Aldose reductase	Akr1b1
Q8N1Q1	Carbonic anhydrase 13	CA13
Q14833	Metabotropic glutamate receptor 4	GRM4
P43681	Neuronal acetylcholine receptor subunit alpha-4	CHRNA4
P30305	M-phase inducer phosphatase 2	CDC25B
Q9Y2D0	Carbonic anhydrase 5B, mitochondrial	CA5B
P22748	Carbonic anhydrase 4	CA4
P23280	Carbonic anhydrase 6	CA6
P05186	Alkaline phosphatase, tissue-nonspecific isozyme	ALPL
Q9NR96	Toll-like receptor 9	TLR9
P37058	Testosterone 17-beta-dehydrogenase 3	HSD17B3
P04150	nuclear receptor subfamily 3 group C member 1	NR3C1
P10275	androgen receptor	AR
O75469	nuclear receptor subfamily 1, group I, member 2	NR1I2
P10828	thyroid hormone receptor beta	THRB
P03372	estrogen receptor 1	ESR1
P04637	tumor protein p53	TP53
P37231	peroxisome proliferator activated receptor	PPARG
Q9NUW8	tyrosyl DNA phosphodiesterase 1	TDP1
Q9NNW7	thioredoxin reductase 2	TXNRD2
Q16881	thioredoxin reductase 1	TXNRD1

Q12809	potassium voltage gated channel subfamily H member 2	KCNH2
P35462	dopamine receptor D3	DRD3
Q96QE3	ATPase family AAA domain containing 5	ATAD5
P35869	aryl hydrocarbon receptor	AHR
P11511	cytochrome P450 family 19 subfamily A member 1	CYP19A1
Q8N884	cyclic GMP AMP synthase	CGAS
P08684	cytochrome P450 family 3 subfamily A member 4	CYP3A4
P42574	Caspase-3	CASP3
P06748	Nucleophosmin 1	NPM1
P49715	Ccaat Enhancer Binding Protein Alpha	CEBPA
P36888	Fms Related Receptor Tyrosine Kinase 3	FLT3
P12004	Proliferating Cell Nuclear Antigen	PCNA
Q13951	Core-Binding Factor Subunit Beta	CBFB
Q16690	Dual Specificity Phosphatase 5	DUSP5
Q06455	Runx1 Partner Transcriptional Co-Repressor 1	RUNXLT1
P35749	Myosin Heavy Chain 11	MYH11
Q8TEK3	Dot1 Like Histone Lysine Methyltransferase	DOT11
P50281	Matrix Metallopeptidase 14	MMP14
Q01196	Runx Family Transcription Factor 1	RUNX1
P30307	Cell Division Cycle 25c	MPIP3
P29590	Pml Nuclear Body Scaffold	PML
Q15910	Enhancer Of Zeste 2 Polycomb Repressive Complex 2 Subunit	EZH2
P20248	Cyclin A2	CCNA2
P06493	Cyclin-Dependent Kinase A-1	CDK1
P24941	Cyclin Dependent Kinase 2	CDK2
P08253	Gelatinase A	MMP2
Q07817	Bcl2 Like 1	B2CL1
P60484	Phosphatase And Tensin Homolog	PTEN
P09874	Poly (Adp-Ribose) Polymerase	PARP1
P01138	Nerve Growth Factor	NGF
Q13470	Tyrosine Kinase Non Receptor 1	TNK1
P10415	Bcl2 Apoptosis Regulator	BCL2
P15692	Vascular Endothelial Growth Factor A	VEGFA
P04040	Catalase	CATA
P31749	Akt Serine/Threonine Kinase 1	AKT1
P51817	Protein Kinase X-Linked	PRKX
