# Supplementary material for: Exploring the Mechanisms of Arsenic Trioxide (Pishuang) in Hepatocellular Carcinoma Based on Network Pharmacology
Source: Evid Based Complement Alternat Med. 2021 Nov 29;2021:5773802. doi: 10.1155/2021/5773802 (PMC8648446; doi:10.1155/2021/5773802)
Supplement: Supplementary Materials — Supplementary Table S1: arsenic trioxide targets. Supplementary Table S2: hepatocellular carcinoma targets. Supplementary Table S3: KEGG pathways (P < 0.05). [file 5773802.f1.zip › 5773802.f1/Supplementary Table S2.pdf]

Supplementary Table S2. Hepatocellular carcinoma targets

Gene Symbol	uniprot ID	Relevance score
BRCA2	P51587	205.549057
BRCA1	P38398	195.1322784
TP53	P04637	179.5122681
APC	P25054	146.0582886
CDH1	P12830	131.5046692
MSH2	P43246	126.5668716
PTEN	P60484	125.4214935
MSH6	P52701	124.9148636
ATM	Q13315	123.2937546
KRAS	P01116	115.3674774
MLH1	P40692	114.2893906
PALB2	Q86YC2	114.1097946
PIK3CA	P42336	111.3293304
CHEK2	O96017	109.5478821
PMS2	P54278	102.531723
CTNNB1	P35222	99.72239685
EGFR	P00533	99.37739563
MET	P08581	99.35324097
BRIP1	Q9BX63	95.58755493
ERBB2	P04626	94.99286652
CDKN2A	P42771	94.94598389
BRAF	P15056	88.77684784
AKT1	P31749	86.17378235
SMAD4	Q13485	85.82302094
NBN	O60934	85.06786346
BARD1	Q99728	84.55039978
POLE	Q07864	84.4451828
STK11	Q15831	82.13739014
TSC2	P49815	79.83319855
MUTYH	Q9UIF7	78.88843536
CASP8	Q14790	78.17385864
RAD51C	O43502	77.62724304
RB1	P06400	77.176651
RAD51D	O75771	74.55080414
RET	P07949	74.15335083
C11orf65	Q8NCR3	73.23046875
DICER1	Q9UPY3	72.19197845
POLD1	P28340	71.97610474
TERT	O14746	71.4797821
AXIN2	Q9Y2T1	71.33855438
TSC1	Q92574	71.17622375
NF1	P21359	70.67160034
CCND1	P24385	68.27788544
ESR1	P03372	65.49526215

EPCAM	P16422	65.2386322
NRAS	P01111	64.50933075
RAD50	Q92878	61.40025711
SMARCA4	P51532	61.39935303
AR	P10275	61.18000793
KIT	P10721	60.55977631
FGFR3	P22607	59.67504883
HRAS	P01112	59.54817581
EGF	P01133	59.40391922
PKHD1	P08F94	58.84106064
FGFR2	P21802	58.0815773
TGFBR2	P37173	57.91071701
PPARG	P37231	57.061409
IGF2	P01344	56.8068428
IL6	P05231	56.73911667
PKD1	P98161	56.68632507
CTNNA1	P35221	56.51578522
PTCH1	Q13635	55.92332458
CDKN1B	P46527	54.3887825
IL1B	P01584	54.34818268
HFE	Q30201	54.04634094
FANCC	Q00597	53.96107864
CDK4	P11802	53.70964432
MLH3	Q9UHC1	53.50416946
ALK	Q9UM73	53.35427856
FASLG	P48023	53.141922
RAD51	Q06609	52.97978973
TNF	P01375	52.40247345
BMPR1A	P36894	52.33736038
SEC63	Q9UGP8	52.09539413
MAP2K1	Q02750	51.73163223
BLM	P54132	51.5787735
MRE11	P49959	51.18395233
XRCC2	O43543	51.08583832
BAX	Q07812	50.86268234
MDM2	Q00987	50.6002388
BAP1	Q92560	50.17434692
VHL	P40337	49.87156677
HNF1B	P35680	49.1841774
HOXB13	Q92826	48.90751266
FH	P07954	48.5976181
TGFB1	P01137	48.18391418
MEN1	O00255	47.75595474
MSH3	P20585	47.66666412
MYC	P01106	46.83638763
SMARCB1	Q12824	46.27189636
PRKCSH	P14314	46.14991379

PIK3R1	P27986	45.72460938
IGF2R	P11717	45.36416626
FLCN	Q8NFG4	45.34634018
SDHB	P21912	45.31417847
EP300	Q09472	45.27028656
KRT18	P05783	45.10536194
AXIN1	O15169	44.94358063
STAT3	P40763	44.63430023
ERCC6	Q03468	44.62662888
CDKN3	Q16667	44.47290802
SRC	P12931	44.46234894
KLF6	Q99612	44.33501053
PDGFRL	Q15198	44.30499268
VEGFA	P15692	43.9585495
JAK2	O60674	43.91513443
AURKA	O14965	43.71445465
DLC1	Q96QB1	42.9414711
KRT8	P05787	42.81023026
PMS1	P54277	41.9646759
ALB	P02768	41.68463516
FGFR4	P22455	41.40167618
SLC22A18	Q96BI1	41.26567841
CDC73	Q6P1J9	40.92840958
PTPN11	Q06124	40.90694427
MTOR	P42345	40.86098862
SDHA	P31040	40.83311462
FAS	P25445	40.65274048
CYP2A6	P11509	40.62194824
PKD2	Q13563	40.51825333
XRCC3	O43542	40.40222549
LRP5	O75197	40.15058899
SDHD	O14521	40.09207916
HNF1A	P20823	39.96606445
IL10	P22301	39.81573868
PRKN	O60260	39.74688721
AFP	P02771	39.55162048
MT-CYB	P00156	39.0334053
MSR1	P21757	38.94449234
MARS1	P56192	38.91863251
SERPINA1	P01009	38.87588501
TRMU	O75648	38.71746826
PRMT7	Q9NVM4	38.64316559
INS	P01308	38.61871719
IL1RN	P18510	38.59367371
IRF1	P10914	38.42256165
FANCM	Q8IYD8	38.30975342
ABCB11	O95342	37.68312454

ABRAXAS1	Q6UWZ7	37.41709518
ABCC2	Q92887	37.40346909
EPHB2	P29323	37.34417725
GSTM1	P09488	37.31334686
LRRC56	Q8IYG6	37.22994614
FBXW7	Q969H0	37.06254578
GANAB	Q14697	36.86803436
NBAS	A2RRP1	36.82516861
PALLD	Q8WX93	36.59095383
CASP3	P42574	36.42987823
CYP3A4	P08684	36.24917221
PDGFRA	P16234	36.22436142
TLR2	O60603	36.17228699
RNASEL	Q05823	35.95724869
BUB1	O43683	35.89767075
CXCL8	P10145	35.7097168
ABCB1	P08183	35.51445389
PPM1D	O15297	35.50196075
HGF	P14210	35.4146347
CDKN1A	P38936	35.35334396
MMP2	P08253	35.1183548
GSTP1	P09211	35.11188507
SDHC	Q99643	35.10485458
PNPLA3	Q9NST1	35.09279633
CYP2E1	P05181	35.06515121
IGF1	P05019	35.00359726
WT1	P19544	34.97868729
SUFU	Q9UMX1	34.85001755
ROS1	P08922	34.70681381
DCC	P43146	34.60736847
NFKB1	P19838	34.22917557
NOTCH1	P46531	34.05355835
MTUS1	Q9ULD2	34.03027725
ARID1A	O14497	34.00561523
KLLN	B2CW77	33.97402954
PTGS2	P35354	33.95723343
MMP9	P14780	33.91516113
ERCC1	P07992	33.83814621
MT-CO1	P00395	33.76021194
ERCC2	P18074	33.72378922
CYP1A1	P04798	33.66807175
ZFHX3	Q15911	33.61841965
GPT	P24298	33.49251938
BCL2	P10415	33.47279739
BUB1B	O60566	33.43701553
UGT1A1	P22309	33.32890701
MCC	P23508	33.2741127

PHB	P35232	33.20516205
ELAC2	Q9BQ52	33.20398712
DLEC1	Q9Y238	33.06842804
HIF1A	Q16665	33.01151657
PPP2R1B	P30154	32.96010971
CTLA4	P16410	32.78451157
MAPK1	P28482	32.73587418
KRT19	P08727	32.64953995
POT1	Q9NUX5	32.63805008
RINT1	Q6NUQ1	32.54071808
IFNG	P01579	32.5286293
SOX9	P48436	32.4931488
GREM1	O60565	32.47205734
RAD54L	Q92698	32.18767166
GPC3	P51654	32.14364624
PHKA2	P46019	32.00918579
IDH1	O75874	31.63043213
POLG	P54098	31.45546341
ABCB4	P21439	31.43757248
MUC1	P15941	31.39294624
SRD5A2	P31213	31.29135704
MAD1L1	Q9Y6D9	31.22601128
JUN	P05412	31.15999985
SMAD7	O15105	30.99428558
NR1H4	Q96RI1	30.98490715
FBN1	P35555	30.69669342
CAV1	Q03135	30.62820053
DMD	P11532	30.58630562
RB1CC1	Q8TDY2	30.51896095
SMARCE1	Q969G3	30.42705917
FHIT	P49789	30.3963604
WWOX	Q9NZC7	30.37452888
TNFRSF10B	O14763	30.34324265
MTHFR	P42898	30.33177948
HNF4A	P41235	30.33177185
MT-CO2	P00403	30.23295975
CD44	P16070	30.14464951
F2	P00734	29.99422073
BCL10	O95999	29.93365288
CASP10	Q92851	29.87865829
NKX2-1	P43699	29.84688377
MAP3K1	Q13233	29.84490013
IL2	P60568	29.83140945
CD274	Q9NZQ7	29.82919502
AKT2	P31751	29.60186005
MAPK8	P45983	29.5794754
BIRC5	O15392	29.56040192

KRT7	P08729	29.55821037
PYGL	P06737	29.53203392
CXCR4	P61073	29.4767189
AIP	O00170	29.46490479
ENG	P17813	29.43889618
MGMT	P16455	29.43452072
ESR2	Q92731	29.39658737
POLK	Q9UBT6	29.39329338
FGFR1	P11362	29.34286499
MSMB	P08118	29.29538155
CEACAM5	P06731	29.2677269
KDR	P35968	29.2488327
AOPEP	Q8N6M6	29.21659088
UCA1	Q2VCE5	29.13513184
GNAS1	Q5JWF2	29.09568405
GNAS2	P63092	
GNAS3	O95467	
XIAP	P98170	29.08225632
RAF1	P04049	29.05905151
RELA	Q04206	29.00323486
IGF1R	P08069	28.93559837
FOS	P01100	28.88524818
ABCG2	Q9UNQ0	28.83055878
SOD2	P04179	28.71426392
RRAS2	P62070	28.6684227
HADHA	P40939	28.61536407
SERPINA3	P01011	28.48402786
ERCC4	Q92889	28.46408081
CYCS	P99999	28.36730957
SLC2A1	P11166	28.36564827
GGT1	P19440	28.29611206
ETV6	P41212	28.29177094
APOE	P02649	28.25263596
STAT1	P42224	28.24811935
EZH2	Q15910	28.19043732
TP73	O15350	28.18159866
PLA2G2A	P14555	28.07972336
NTHL1	P78549	28.03541565
MMP1	P03956	27.97943497
RUNX1	Q01196	27.9661293
CYP1A2	P05177	27.93668365
CXCL12	P48061	27.93318939
GYS2	P54840	27.91932678
IDH2	P48735	27.89899254
HMMR	O75330	27.89712334
EHBP1	Q8NDI1	27.80945778
IGFBP3	P17936	27.80731583

CPT1A	P50416	27.70864296
WRAP53	Q9BUR4	27.64285278
MT-ND1	P03886	27.60913658
ATP7B	P35670	27.49726868
SPP1	P10451	27.40171051
RASSF1	Q9NS23	27.32366753
CTAG1B	P78358	27.28216934
GALNT12	Q8IXK2	27.26653671
TYMP	P19971	27.25923157
BCL2L1	Q07817	27.25351334
MXRA5	Q9NR99	27.19938469
PLAU	P00749	27.19470215
PGR	P06401	27.03931999
SMAD3	P84022	27.02907372
HLA-DRB1	P01911	27.02516556
ATR	Q13535	27.00310326
MAP3K8	P41279	26.92336273
FOXP3	Q9BZS1	26.889431
NQO2	P16083	26.86259651
TLR4	O00206	26.84511948
ITGB1	P05556	26.76062012
MAX	P61244	26.72741318
MXI1	P50539	26.64262009
TNFSF10	P50591	26.609375
JAG1	P78504	26.58377838
TMEM127	O75204	26.55732536
FARSB	Q9NSD9	26.50778198
IRS1	P35568	26.42150307
FGF2	P09038	26.4119091
DDB2	Q92466	26.38834763
CRP	P02741	26.38308716
ABL1	P00519	26.35638046
PHKG2	P15735	26.333395
PGBD3	Q8N328	26.28913116
PDGFRB	P09619	26.25417709
NOTCH2	Q04721	26.24195099
DNMT1	P26358	26.23770714
PRKAR1A	P10644	26.21893692
TYMS	P04818	26.21094894
FASN	P49327	26.2051239
ADIPOQ	Q15848	26.19914246
ALDH2	P05091	26.14962387
NQO1	P15559	26.1333828
MYCN	P04198	26.09466553
NAT2	P11245	26.07409859
ATP8B1	O43520	26.06010818
G6PC1	P35575	26.05454254

PROM1	O43490	26.03831863
RBBP8	Q99708	26.00697899
TIMP1	P01033	25.98859787
PTPRJ	Q12913	25.97432518
LEP	P41159	25.94664001
FABP1	P07148	25.88144302
CASP9	P55211	25.80403519
ALG9	Q9H6U8	25.75902557
MITF	O75030	25.73189735
HERC2	O95714	25.55633545
TGFA	P01135	25.46498871
PTK2	Q05397	25.43745804
RAD54B	Q9Y620	25.35171509
PARP1	P09874	25.33227158
CYP2D6	P10635	25.30657005
INSR	P06213	25.20582962
NFKBIA	P25963	25.18939972
CDK2	P24941	25.17791367
NFE2L2	Q16236	25.04854965
MMP7	P09237	25.03469086
PRKCD	Q05655	25.0200882
TCF7L2	Q9NQB0	24.99434662
NF2	P35240	24.96480751
XRCC1	P18887	24.94275856
NTRK1	P04629	24.90656662
CASC2	Q8IU53	24.8337841
CYP19A1	P11511	24.77781105
CDK1	P06493	24.76931763
RECQL	P46063	24.68499374
CDKN2B	P42772	24.66004753
MAPK3	P27361	24.63134003
TGFBR1	P36897	24.63095474
TWIST1	Q15672	24.57004166
FLT1	P17948	24.51514053
ABCC1	P33527	24.51171494
LARS1	Q9P2J5	24.5049839
NR1H2	P55055	24.49567604
YAP1	P46937	24.45713425
CD34	P28906	24.41926193
PDGFB	P01127	24.402174
SP1	P08047	24.37969971
NOS2	P35228	24.3716507
ERBB3	P21860	24.23073769
KLK3	P07288	24.22393417
SLCO1B1	Q9Y6L6	24.18742752
TOP2A	P11388	24.15598869
MCL1	Q07820	24.14984894

PTPN12	Q05209	24.1051445
UGT1A7	Q9HAW7	24.06089401
SLC17A5	Q9NRA2	24.02213097
NR1H3	Q13133	23.94052315
APOB	P04114	23.93871307
RNF43	Q68DV7	23.85346985
SETD2	Q9BYW2	23.84964943
HSPB1	P04792	23.83499527
GLI1	P08151	23.79854584
SLC25A13	Q9UJS0	23.73480606
AKT3	Q9Y243	23.69870377
VIM	P08670	23.6875267
GSK3B	P49841	23.6817894
FANCI	Q9NVI1	23.67895699
SERPINE1	P05121	23.66568756
DPYD	Q12882	23.66213226
MMP14	P50281	23.65587807
MKI67	P46013	23.62345695
E2F1	Q01094	23.61639023
IFNA1	P01562	23.61079788
CCR6	P51684	23.59442139
FLT3	P36888	23.5786171
CDK6	Q00534	23.55461502
HMOX1	P09601	23.54436111
PDCD1	Q15116	23.53666496
PRKCA	P17252	23.52952576
JAK1	P23458	23.50571442
BMP6	P22004	23.47459793
BMP2	P12643	23.44394302
DNMT3A	Q9Y6K1	23.4011116
VEGFC	P49767	23.36071777
TCF4	P15884	23.33955765
SLMAP	Q14BN4	23.26535225
OGG1	O15527	23.26528358
IFNA2	P01563	23.22385979
PPARA	Q07869	23.21972656
CCNA2	P20248	23.17456436
LZTS1	Q9Y250	23.16333008
SMAD2	Q15796	23.15408134
ALG8	Q9BVK2	23.11328697
ACTB	P60709	23.08187294
PLAUR	Q03405	23.04400063
CEBPA	P49715	22.96249008
CCL2	P13500	22.93404007
TIMP2	P16035	22.92636299
AMER1	Q5JTC6	22.91539574
AMACR	Q9UHK6	22.87238693

MT-ND4L	P03901	22.8486805
SDHAF2	Q9NX18	22.81300926
FN1	P02751	22.79556465
MAPK14	Q16539	22.79017067
APOA1	P02647	22.7351265
PCNA	P12004	22.71648216
HAMP	P81172	22.7078476
GFER	P55789	22.67945862
HLA-DQB1	P01920	22.64009476
VDR	P11473	22.60383415
ARG1	P05089	22.57094574
MAP3K6	O95382	22.51157761
LEPR	P48357	22.4837265
ING1	Q9UK53	22.41225815
CFTR	P13569	22.38201904
MYO1B	O43795	22.34225845
FAH	P16930	22.27680206
RARB	P10826	22.24311829
NPM1	P06748	22.22330666
ZEB1	P37275	22.17970276
H2AC18	Q6FI13	22.15608978
CDKN1C	P49918	22.15252495
OPCML	Q14982	22.13502312
GSTT1	P30711	22.12074661
TGFB2	P61812	22.10462761
SNAI1	O95863	22.09888077
ALDOB	P05062	22.07601929
PTPRC	P08575	22.06052971
FOXM1	Q08050	22.04136276
CP	P00450	21.97289085
TRIP13	Q15645	21.97002411
MUC16	Q8WXI7	21.96983528
HSP90AA1	P07900	21.89158058
RHOA	P61586	21.86969566
RPS6KB1	P23443	21.84831238
TP63	Q9H3D4	21.79339409
DROSHA	Q9NRR4	21.78801727
PFKL	P17858	21.7787838
PIK3CG	P48736	21.76105881
CDH17	Q12864	21.70757675
SQSTM1	Q13501	21.69415283
ENO2	P09104	21.66785431
XPA	P23025	21.6665535
SNAI2	O43623	21.66382217
CDH2	P19022	21.65257835
TJP2	Q9UDY2	21.58202553
HSPA5	P11021	21.56615639

FAN1	Q9Y2M0	21.53215218
GAPDH	P04406	21.4825058
SST	P61278	21.4488678
CREBBP	Q92793	21.41208458
NME1	P15531	21.38324356
INS-IGF2	F8WCM5	21.38170242
CSF3	P09919	21.37967682
XBP1	P17861	21.37768173
CCNE1	P24864	21.37665558
CCNB1	P14635	21.37563705
IL1A	P01583	21.37197876
DHFR	P00374	21.36401367
ICAM1	P05362	21.33664703
HDAC1	Q13547	21.24301147
CHGA	P10645	21.21774292
SIRT1	Q96EB6	21.19445801
SOS1	Q07889	21.17912483
NPC1	O15118	21.15859985
IL4	P05112	21.15553284
DNMT3B	Q9UBC3	21.01635551
ABCA1	O95477	20.99132919
IRS2	Q9Y4H2	20.95711899
ANXA5	P08758	20.95506096
SLC9A9	Q8IVB4	20.85512924
SLCO1B3	Q9NPD5	20.85288239
SOCS3	O14543	20.79307175
DNAJB11	Q9UBS4	20.74754906
TNFRSF1A	P19438	20.69101334
ABCC3	O15438	20.673172
UGT1A6	P19224	20.65515137
TTR	P02766	20.64532852
SPINK1	P00995	20.60514832
BAK1	Q16611	20.59474373
HSPA4	P34932	20.56010056
TF	P02787	20.52710342
FZD3	Q9NPG1	20.4777298
IL6R	P08887	20.44932938
CYP1B1	Q16678	20.44902802
THPO	P40225	20.4149971
FLT4	P35916	20.41319656
EPHX1	P07099	20.40729713
RAC1	P63000	20.40197945
TFR2	Q9UP52	20.3974762
CPT2	P23786	20.33948898
GNAQ	P50148	20.33937073
TET2	Q6N021	20.33860779
ERCC3	P19447	20.25450325

HABP2	Q14520	20.23817825
RXRA	P19793	20.21387482
CALR	P27797	20.20253563
ALPP	P05187	20.12555695
ASS1	P00966	20.11824226
HADHB	P55084	20.06916428
IL2RA	P01589	20.01998901
