# Supplementary material for: Exploring the Mechanisms of Arsenic Trioxide (Pishuang) in Hepatocellular Carcinoma Based on Network Pharmacology
Source: Evid Based Complement Alternat Med. 2021 Nov 29;2021:5773802. doi: 10.1155/2021/5773802 (PMC8648446; doi:10.1155/2021/5773802)
Supplement: Supplementary Materials — Supplementary Table S1: arsenic trioxide targets. Supplementary Table S2: hepatocellular carcinoma targets. Supplementary Table S3: KEGG pathways (P < 0.05). [file 5773802.f1.zip › 5773802.f1/Supplementary Table S3.pdf]

Supplementary Table S3. KEGG pathways (P＜0.05)

Term	PValue	Genes	Count
hsa05200:Pathways in cancer	2.14E-17	CEBPA, GSK3B, FLT3, HDAC1, PTEN, PTGS2, RELA, CASP9, CASP3, AKT1, RAC1, HSP90AA1, NOS2, MMP2, MMP9, RUNX1, VEGFA, AR, CDK2, BCL2,	25
hsa05215:Prostate cancer	1.97E-11	CASP9, GSK3B, AR, HSP90AA1, SRD5A2, CDK2, PTEN, BCL2, AKT1,	12
hsa05222:Small cell lung cancer	3.32E-10	CASP9, NOS2, CDK2, PTEN, BCL2, RARB, AKT1, PTGS2, TP53, RELA,	11
hsa05161:Hepatitis B	4.61E-09	CASP9, CCNA2, PCNA, CASP3, CDK2, PTEN, BCL2, AKT1, RAF1, TP53, MMP9,	12
hsa04151:PI3K-Akt signaling pathway	1.07E-08	GSK3B, HSP90AA1, PTEN, RELA, VEGFA, CASP9, RPS6KB1, CDK2, BCL2, AKT1, RAC1, JAK2, RAF1, TP53, MCL1,	16
hsa05206:MicroRNAs in cancer	7.52E-08	DNMT1, ABCB1, PTEN, PTGS2, SIRT1, MMP9, VEGFA, PLAU, CASP3, BCL2, RAF1, TP53, EZH2, MCL1	14
hsa05210:Colorectal cancer	2.52E-07	CASP9, GSK3B, CASP3, BCL2, AKT1, RAC1, RAF1, TP53	8
hsa05212:Pancreatic cancer	3.51E-07	CASP9, AKT1, RAC1, RAF1, TP53, RELA, BCL2L1, VEGFA	8
hsa05205:Proteoglycans in cancer	1.28E-06	RPS6KB1, PLAU, CASP3, MMP2, AKT1, RAC1, RAF1, ESR1, TP53, MMP9,	11
hsa05202:Transcriptional misregulation in cancer	2.52E-06	CEBPA, PLAU, FLT3, HDAC1, PPARG, TP53, MMP9, RELA, BCL2L1, RUNX1	10
hsa05221:Acute myeloid leukemia	2.67E-06	CEBPA, RPS6KB1, FLT3, AKT1, RAF1, RELA, RUNX1	7
hsa04210:Apoptosis	4.89E-06	CASP9, CASP3, BCL2, AKT1, TP53, RELA, BCL2L1	7
hsa04917:Prolactin signaling	1.08E-05	GSK3B, AKT1, JAK2, RAF1, ESR1,	7
hsa05220:Chronic myeloid leukemia	1.18E-05	HDAC1, AKT1, RAF1, TP53, RELA, BCL2L1, RUNX1	7
hsa05145:Toxoplasmosis	1.25E-05	CASP9, NOS2, CASP3, BCL2, AKT1, JAK2, RELA, BCL2L1	8
hsa05014:Amyotrophic lateral sclerosis (ALS)	2.74E-05	CASP9, CASP3, BCL2, RAC1, TP53, BCL2L1	6
hsa05213:Endometrial cancer	3.32E-05	CASP9, GSK3B, PTEN, AKT1, RAF1,	6
hsa04914:Progesterone-mediated oocyte maturation	3.50E-05	CCNA2, HSP90AA1, CDK2, CDK1, AKT1, PGR, RAF1	7
hsa04370:VEGF signaling	7.24E-05	CASP9, AKT1, RAC1, RAF1, PTGS2,	6
hsa04915:Estrogen signaling pathway	7.27E-05	HSP90AA1, MMP2, AKT1, RAF1, ESR1, MMP9, ESR2	7
hsa04115:p53 signaling	1.14E-04	CASP9, CASP3, CDK2, PTEN, CDK1,	6
hsa04919:Thyroid hormone signaling pathway	1.67E-04	CASP9, GSK3B, HDAC1, AKT1, RAF1, ESR1, TP53	7
hsa05219:Bladder cancer	2.05E-04	MMP2, RAF1, TP53, MMP9, VEGFA	5

hsa04722:Neurotrophin signaling pathway	2.11E-04	GSK3B, BCL2, AKT1, RAC1, RAF1, TP53, RELA	7
hsa04071:Sphingolipid signaling pathway	2.11E-04	PTEN, BCL2, AKT1, RAC1, RAF1, TP53, RELA	7
hsa05169:Epstein-Barr virus infection	2.31E-04	CCNA2, HDAC1, CDK2, BCL2, AKT1, TP53, RELA	7
hsa04110:Cell cycle	2.52E-04	CCNA2, GSK3B, PCNA, HDAC1, CDK2, CDK1, TP53	7
hsa05152:Tuberculosis	2.60E-04	CASP9, NOS2, CASP3, BCL2, AKT1, JAK2, RAF1, RELA	8
hsa04066:HIF-1 signaling	6.15E-04	RPS6KB1, NOS2, BCL2, AKT1, RELA,	6
hsa05203:Viral carcinogenesis	6.34E-04	CCNA2, HDAC1, CASP3, CDK2, CDK1, RAC1, TP53, RELA	8
hsa05223:Non-small cell lung	6.87E-04	CASP9, RARB, AKT1, RAF1, TP53	5
hsa04932:Non-alcoholic fatty liver disease (NAFLD)	7.25E-04	GSK3B, CEBPA, CASP3, NR1H3, AKT1, RAC1, RELA	7
hsa04668:TNF signaling	0.001007	MMP14, CASP3, AKT1, PTGS2, MMP9,	6
hsa04931:Insulin resistance	0.00105	GSK3B, RPS6KB1, PTEN, NR1H3, AKT1,	6
hsa05230:Central carbon metabolism in cancer	0.001139	FLT3, PTEN, AKT1, RAF1, TP53	5
hsa04662:B cell receptor signaling pathway	0.001509	GSK3B, AKT1, RAC1, RAF1, RELA	5
hsa04152:AMPK signaling	0.001876	CCNA2, RPS6KB1, HNF4A, AKT1,	6
hsa05160:Hepatitis C	0.002643	GSK3B, NR1H3, AKT1, RAF1, TP53,	6
hsa05162:Measles	0.002643	GSK3B, CDK2, AKT1, JAK2, TP53, RELA	6
hsa04666:Fc gamma R-mediated phagocytosis	0.003109	PTPRC, RPS6KB1, AKT1, RAC1, RAF1	5
hsa04064:NF-kappa B signaling pathway	0.00353	PLAU, BCL2, PTGS2, RELA, BCL2L1	5
hsa04510:Focal adhesion	0.00355	GSK3B, PTEN, BCL2, AKT1, RAC1,	7
hsa04660:T cell receptor signaling pathway	0.005803	GSK3B, PTPRC, AKT1, RAF1, RELA	5
hsa05164:Influenza A	0.008255	CASP9, GSK3B, AKT1, JAK2, RAF1,	6
hsa00140:Steroid hormone biosynthesis	0.008421	SRD5A2, CYP1A2, CYP3A4, CYP19A1	4
hsa05166:HTLV-I infection	0.00972	GSK3B, PCNA, TERT, AKT1, TP53, RELA, BCL2L1	7
hsa05168:Herpes simplex	0.010147	CASP3, CDK2, CDK1, JAK2, TP53, RELA	6
hsa04062:Chemokine signaling pathway	0.010838	GSK3B, AKT1, RAC1, JAK2, RAF1, RELA	6
hsa05214:Glioma	0.011506	PTEN, AKT1, RAF1, TP53	4
hsa05211:Renal cell	0.011993	AKT1, RAC1, RAF1, VEGFA	4
hsa05140:Leishmaniasis	0.014605	NOS2, JAK2, PTGS2, RELA	4
hsa05218:Melanoma	0.014605	PTEN, AKT1, RAF1, TP53	4
hsa04068:FoxO signaling	0.015874	CDK2, PTEN, AKT1, RAF1, SIRT1	5
hsa04014:Ras signaling	0.023347	AKT1, RAC1, RAF1, RELA, BCL2L1,	6
hsa04012:ErbB signaling	0.024986	GSK3B, RPS6KB1, AKT1, RAF1	4
hsa04010:MAPK signaling	0.035661	CASP3, AKT1, RAC1, RAF1, TP53, RELA	6

hsa05231:Choline metabolism in cancer	0.036618	RPS6KB1, AKT1, RAC1, RAF1	4
hsa04114:Oocyte meiosis	0.04636	AR, CDK2, CDK1, PGR	4
hsa04726:Serotonergic	0.04636	CYP2D6, CASP3, RAF1, PTGS2	4
